# Supplementary material for: Antibacterial Activity of ZnO Nanoparticles in a Staphylococcus-aureus-Infected Galleria mellonella Model Is Tuned by Different Apple-Derived Phytocargos
Source: J Funct Biomater. 2023 Sep 8;14(9):463. doi: 10.3390/jfb14090463 (PMC10532052; doi:10.3390/jfb14090463)
Supplement: Supplementary file 1 [file jfb-14-00463-s001.zip › jfb-2514262-supplementary.pdf]

*Article*

# Antibacterial Activity of ZnO Nanoparticles in a *Staphylococcus-aureus*-Infected *Galleria mellonella* Model Is Tuned by Different Apple-Derived Phytocargos

**Catarina F. Santos[1,2,*], Suzana M. Andrade[2], Dalila Mil-Homens[3,4], M. Fátima Montemor[2], Marta M. Alves[2,*]**

[1] EST Setúbal, CDP2T, Instituto Politécnico de Setúbal, Campus IPS, 2910 Setúbal, Portugal.

[2] Centro de Química Estrutural (CQE), Institute of Molecular Sciences, Molecular Departamento de Engenharia Química (DEQ), Instituto Superior Técnico, Universidade de Lisboa, Av. Rovisco Pais 1049-001, Lisboa, Portugal

[3] iBB-Institute for Bioengineering and Biosciences, and i4HB-Institute for Health and Bioeconomy, Instituto Superior Técnico, Universidade de Lisboa, Av. Rovisco Pais 1049-001 Lisboa, Portugal.

[4] Department of Bioengineering, Instituto Superior Técnico, Universidade de Lisboa, Av. Rovisco Pais 1049-001 Lisboa, Portugal.

* Correspondence: catarina.santos@estsetubal.ips.pt (CFS); martamalves@tecnico.ulisboa.pt (MMA)

**Supplementary Information**

## Results and discussion

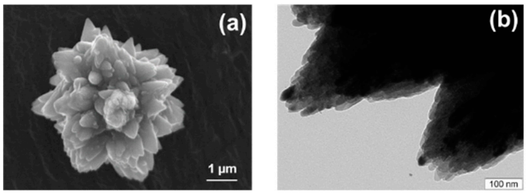


**Figure S1** – Morphological characterization of ZnO particles synthesized without phytochemicals; (a) scanning electron microscopy (SEM) and (b) transmission electron microscopy images of ZnO (without phytocargo) [1,2].

**Table S1.** Chemical elemental quantification made by EDS

| | **BE-ZnO8** | **BE-ZnO12** |
|---|---|---|
| **Element** | **At %** | **At %** |
| C 1s | 39.5 | 50.7 |
| O 1s | 47.9 | 40.0 |
| Zn 2p3 | 12.7 | 9.3 |

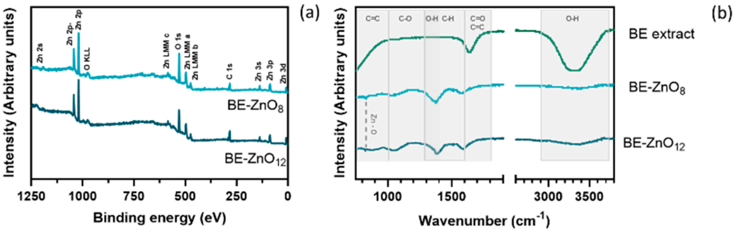


**Figure S2.** Chemical analysis of BE-ZnO NPs and *'Bravo de Esmolfe'* apple extract; (a) X-ray photoelectron spectroscopy (XPS) survey spectra for BE-ZnO8 and BE-ZnO12 NPs; (b) attenuated total reflection-Fourier transform infrared spectroscopy (ATR-FTIR) of the apple extract and BE-ZnO8 and BE-ZnO12 NPs.

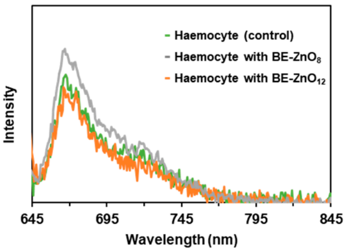


**Figure S3.** Characterisation of Alexa 633 stained haemocytes interaction with BE-ZnO particles; fluorescence spectra of haemocytes without or with BE-ZnO particles.

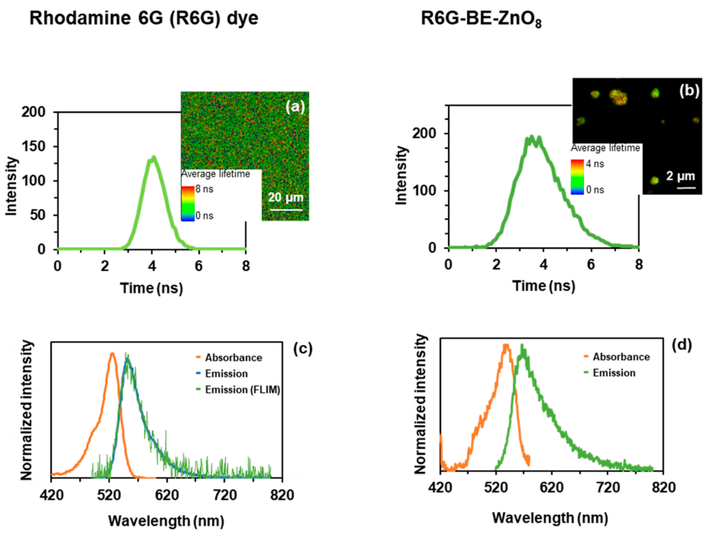


**Figure S4.** Photophysical characterisation of the R6G alone, within BE-$ZnO_8$ particles (R6G-BE-$ZnO_8$); FLIM image and associated histogram of (a) R6G in PBS and (b) R6G-BE-$ZnO_8$ particles. Absorption and emission spectra of (c) R6G in PBS, pH=8 and (d) R6G-BE-$ZnO_8$. The emission spectrum in (c) was also obtained using the FLIM set-up to validate data with the larvae model.

Spectroscopic and morphologic characterisation of the system R6G-BE-$ZnO_8$ (Figure S5b) was carried out using R6G in PBS as a comparison (Figure S5a). Both absorbance and fluorescence emission peaks of R6G (Figure S5c) are red-shifted when associated with BE-$ZnO_8$ particles (Figure S5d), i.e., emission shifts from 555 nm in PBS to 565 nm (Figure S5). It is known that metallic structures can alter the optical properties of adsorbed dyes. Both quenching and enhancement of the fluorescence intensity of the dye have been proposed for interactions with Au nanoparticles (NP) depending, among other factors, on the distance between dye-metal NP [3]. FLIM images provide evidence for R6G-BE-$ZnO_8$ particles. A good dispersion of bright round-shaped nanometric objects can be observed in Figure S5b). From the corresponding FLIM images' histogram (Figure S5a,b), one can denote a strong decrease in the dye fluorescence lifetime, from 4 ns in PBS (Figure S5a) to around 3 ns when in association with the particles (Figure S5b), which points to a short distance between the dye and metal surface. Therefore, our dye-BE-ZnO NP system proved adequate to assess their presence in larvae haemocytes.

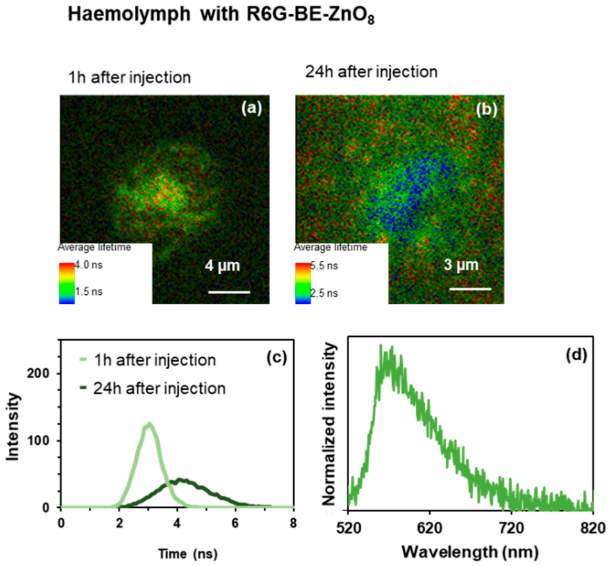


**Figure S5**. Photophysical characterisation of *in vivo* in injected larvae haemolymph and haemocytes: (a, b) FLIM images and (c) associated histogram, and (d) fluorescence spectrum obtained from a bright region in a); $\lambda_{exc}$=483 nm.

To assess the presence of the R6G-BE-$ZnO_8$ particles in the larvae haemocytes upon injection, a screen of the haemolymph (Figure S6a-d), revealed that the signal in the FLIM image was on a haemocyte-like structure 1h after the injection (Figure S6a), whereas 24h after spreading of the signal was already visible in the haemolymph (Figure S6b). The histogram further assessed the presence of the R6G-BE-$ZnO_8$ particles in the haemocytes-like structures, where the expected 3 ns lifetime was depicted for the first hour and a 4 ns lifetime 24 h later (Figure S6c). The fluorescence spectrum obtained from a bright image region in Figure S5d), i.e. from the haemolymph of larvae injected with R6G-BE-$ZnO_8$, supports the adequacy of our system, as the fluorescence signal of the tag particles remains unaltered when in contact with the haemolymph.

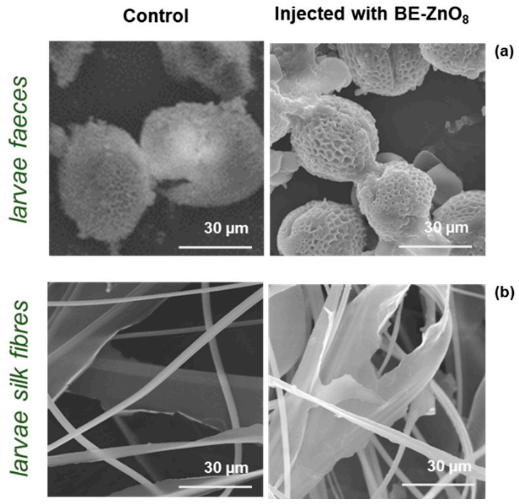


**Figure S6.** Morphological analyses of the (a) faeces and (b) silk fibres of larvae 48h after the injection of PBS (control) (images on the left) and with BE-$ZnO_8$ particles (images on the right).

SEM morphological characterization of larvae excrement and fibres revealed no differences between larvae with BE-$ZnO_8$ particles and control with PBS (Figure S7a and Figure S7b). Aside from that, we can notice the typical form of larvae excrement and fibres [4,5].

## Materials and methods

*Physicochemical characterisation of ZnO particles:* The morphology of the particles was characterised by scanning electron microscopy (SEM) using a JEOL-JSM7001F apparatus, and the elemental chemical composition was assessed by the corresponding X-ray energy dispersive spectrometer (EDS). A thin coating of conductive gold/palladium (Polaron E-5100) was added prior to sample analysis to increase conductivity. For the transmission electron microscopy (TEM) studies, a Hitachi H-9000-NA microscope operated at 200 kV with supporting copper grids was used. The crystallinity was assessed by X-ray diffraction (XRD) using a D8 Advance Bruker AXS θ-2θ diffractometer with a copper radiation source (Cu K$\alpha$, $\lambda$ = 1.5406 Å) and a secondary monochromator operated at 40 kV and 40 mA.

X-ray photoelectron spectroscopy (XPS, Kratos Axis Ultra HSA) with a monochromatic Al source operating at 90 W was employed for chemical identification. XPS spectra were corrected considering the C 1s photoelectron line at 285.0 eV. Using a Shirley background, the spectra obtained were fitted using CASA XPS software (CasaXPS Version 2.3.22PR1.0).

The characterization of the organic groups present in the BE-ZnO NPs, and also in apple extract was made by attenuated total reflectance (ATR), acquired by using a Nicolet (Thermo Electron) spectrometer.

The UV–Vis absorption spectra were recorded with a Perkin Elmer Lambda 35 spectrophotometer. The photoluminescence (PL) spectra were recorded at room temperature with an SPEX Fluorolog (Horiba Jobin Yvon) spectrofluorometer.

Fluorescence lifetime imaging microscopy (FLIM) measurements were performed on a time-resolved confocal fluorescence microscope (MicroTime 200, PicoQuant GmbH) [6] and SymPhoTime software, ver. 5.3.2.2 (PicoQuant GmbH) was used for data acquisition and analysis. Luminescence spectra were obtained utilising a low light-level spectrometer, QE Pro (Ocean Optics), and fibre coupled to the confocal microscope. Excitation was achieved using pulsed laser diode heads (405 nm, 485 nm, 639 nm) at repetition rates of 20/40 MHz. A bandpass filter centred at 695 nm and a transmission window of 55 nm was used for the red diode laser, and a long-pass filter with transmission above 510 nm was used for the remainder diode lasers. Permanent online analysis of the back-reflected and backscattered excitation light was achieved with a CCD camera, which monitors the image seen by the objective. The fluorescence lifetimes were detected with a single-photon-counting avalanche diode (SPAD) (PerkinElmer) whose signal was processed by a TimeHarp 200 TC-SPC PC board (PicoQuant) working in time-tagged time-resolved (TTTR) operation mode. For point-by-point measurements, fluorescence decays of > 30-pixel points were collected.

*Minimum Inhibitory Concentration (MIC):* The MIC was determined by a standard broth microdilution procedure recommended by the

Clinical and Laboratory Standards Institute (CLSI) [7]. The assay was performed using the Staphylococcus aureus methicillin-resistant (MRSA) strain JE2 [8]. Bacterial suspensions were prepared on Mueller Hinton Broth (MHB) (BD Difco, Franklin Lakes, NJ, USA) from centrifuged overnight cultures grown in Tryptic soy broth medium (TSB, BD Difco, Franklin Lakes, NJ, USA) at 37 ºC 120 rpm to a final concentration of 1x106 CFU/ml. A 100 µl of bacterial suspension was added to a sterile 96-well microtiter polypropylene plate containing 100 µL of two-fold dilutions of each ZnO-NPs (ranging between 5 and 0.0098 mg/ml) in MHB. The 96-well plate was incubated at 37°C for 24 h and the MIC was defined as the lowest ZnO-NPs concentration required to inhibit visible bacterial growth. A control without ZnO-NPs was included. The assay was performed in triplicate.

*Minimal bactericidal concentration (MBC):* The minimal bactericidal concentration (MBC) was determined by a colony count procedure recommended by the Clinical and Laboratory Standards Institute (CLSI) [9]. After the MIC assay, aliquots were removed from the wells with no visible bacterial growth, serially diluted in MHB, plated on Tryptic soy agar (TSA) (BD Difco), and incubated at 37°C for 24 h. After incubation, bacterial colonies were counted, and the MBC was defined as the lowest ZnO-NPs concentration that caused ≥99.9% cell death of the initial bacterial inoculum (control without ZnO-NPs). The assay was performed in triplicate.

**References**


1. Alves, M.M.; Andrade, S.M.; Grenho, L.; Fernandes, M.H.; Santos, C.; Montemor, M.F. Influence of apple phytochemicals in ZnO nanoparticles formation, photoluminescence and biocompatibility for biomedical applications. *Materials Science and Engineering: C* **2019**, *101*, 76-87, doi:https://doi.org/10.1016/j.msec.2019.03.084.
2. Soliman, M.M.A.; Alegria, E.C.B.A.; Ribeiro, A.P.C.; Alves, M.M.; Saraiva, M.S.; Fátima Montemor, M.; Pombeiro, A.J.L. Green synthesis of zinc oxide particles with apple-derived compounds and their application as catalysts in the transesterification of methyl benzoates. *Dalton Transactions* **2020**, *49*, 6488-6494, doi:10.1039/d0dt01069c.
3. Andrade, S.M.; Bueno-Alejo, C.J.; Serra, V.V.; Rodrigues, J.M.M.; Neves, M.G.P.M.S.; Viana, A.S.; Costa, S.M.B. Anchoring of Gold Nanoparticles on Graphene Oxide and Noncovalent Interactions with Porphyrinoids. *ChemNanoMat* **2015**, *1*, 502-510, doi:https://doi.org/10.1002/cnma.201500133.
4. Mil-Homens, D.; Barahona, S.; Moreira, R.N.; Silva, I.J.; Pinto, S.N.; Fialho, A.M.; Arraiano, C.M. Stress response protein BolA influences fitness and promotes *Salmonella enterica* Serovar Typhimurium virulence. *Applied and Environmental Microbiology* **2018**, *84*, e02850-02817, doi:doi:10.1128/AEM.02850-17.
5. Dustmann, J., H.; von der Ohe, K. Scanning electron microscopic studies on pollen from honey. IV. Surface pattern of pollen of *Sapium sebiferum* and *Euphorbia* spp (Euphorbiaceae). *Apidologie* **1993**, *24*, 59-66.
6. Andrade, S.M.; Raja, P.; Saini, V.K.; Viana, A.S.; Serp, P.; Costa, S.M.B. Polyelectrolyte-Assisted Noncovalent Functionalization of Carbon Nanotubes with Ordered Self-Assemblies of a Water-Soluble Porphyrin. *ChemPhysChem* **2012**, *13*, 3622-3631, doi:https://doi.org/10.1002/cphc.201200428.

7. (CLSI), C.a.L.S.I. Methods for Dilution Antimicrobial Susceptibility Tests for Bacteria that Grow Aerobically, Approved Standard. M07. 11th ed. Clinical and Laboratory Standards Institute; Wayne, PA, USA. **2018**.
8. Kennedy, A.D.; Otto, M.; Braughton, K.R.; Whitney, A.R.; Chen, L.; Mathema, B.; Mediavilla, J.R.; Byrne, K.A.; Parkins, L.D.; Tenover, F.C.; et al. Epidemic community-associated methicillin-resistant *Staphylococcus aureus*: Recent clonal expansion and diversification. *Proceedings of the National Academy of Sciences* **2008**, *105*, 1327-1332, doi:10.1073/pnas.0710217105.
9. (CLSI), C.a.L.S.I. *Methods for Determining Bactericidal Activity of Antimicrobial Agents; Approved Guidelines. M26-A.Clinical and Laboratory Standards Institute; Wayne, PA, USA.*; 1999.
